# The Protective Effects of Shengmai Formula Against Myocardial Injury Induced by Ultrafine Particulate Matter Exposure and Myocardial Ischemia are Mediated by the PI3K/AKT/p38 MAPK/Nrf2 Pathway

**DOI:** 10.3389/fphar.2021.619311

**Published:** 2021-03-08

**Authors:** Lina Chen, Yuan Guo, Shuiqing Qu, Kai Li, Ting Yang, Yuanmin Yang, Zhongyuan Zheng, Hui Liu, Xi Wang, Shuoqiu Deng, Yu Zhang, Xiaoxin Zhu, Yujie Li

**Affiliations:** ^1^ Artemisinin Research Center, China Academy of Chinese Medical Sciences, Beijing, China; ^2^ Institute of Chinese Materia Medica, China Academy of Chinese Medical Sciences, Beijing, China

**Keywords:** Shengmai formula, myocardial injury, ultrafine particulate matter, oxidative stress, PI3K/AKT/p38 MAPK/Nrf2 pathway

## Abstract

**Background and Purpose: **Ultrafine particulate matter (UFPM) induces oxidative stress (OS) and is considered to be a risk factor of myocardial ischemia (MI). Shengmai formula (SMF) is a traditional Chinese medicine with antioxidant properties and has been used to treat cardiovascular diseases for a long time. The aim of this study was to explore the protective role of SMF and the mechanism by which it prevents myocardial injury in UFPM-exposed rats with MI.

**Methods: **An MI rat model was established. Animals were randomly divided into five groups: sham, UFPM + MI, SMF (1.08 mg/kg⋅d) + UFPM + MI, SMF (2.16 mg/kg⋅d) + UFPM + MI, and SMF (4.32 mg/kg⋅d) + UFPM + MI. SMF or saline was administrated 7 days before UFPM instillation (100 μg/kg), followed by 24 h of ischemia. Physiological and biochemical parameters were measured, and histopathological examinations were conducted to evaluate myocardial damage. We also explored the potential mechanism of the protective role of SMF using a system pharmacology approach and an *in vitro* myoblast cell model with small molecule inhibitors.

**Results: **UFPM produced myocardial injuries on myocardial infarct size; serum levels of LDH, CK-MB, and cardiac troponin; and OS responses in the rats with MI. Pretreatment with SMF significantly attenuated these damages *via* reversing the biomarkers. SMF also improved histopathology induced by UFPM and significantly altered the PI3K/AKT/MAPK and OS signaling pathways. The expression patterns of *Cat*, *Gstk1*, and *Cyba* in the UFPM model group were reversed in the SMF-treated group. In *in vitro* studies, SMF attenuated UFPM-induced reactive oxygen species production, mitochondrial damage, and OS responses. The PI3K/AKT/p38 MAPK/Nrf2 pathway was significantly changed in the SMF group compared with that in the UFPM group, whereas opposite results were obtained for pathway inhibition.

**Conclusion: **These findings indicate that SMF prevents OS responses and exerts beneficial effects against myocardial injury induced by UFPM + MI in rats. Furthermore, the PI3K/AKT/p38 MAPK/Nrf2 signaling pathway might be involved in the protective effects of SMF.

## Introduction

Particulate matter (PM) is a complex mixture composed of coarse particles (diameter, 2.5–10 μm; PM_2.5–10_), fine particles (diameter, ≤2.5 µm; PM_2.5_), and ultrafine particles (diameter, ≤0.1 µm; PM_0.1_ or UFPM). PM is considered an environmental risk factor and a life-threatening public health challenge for humans; PM pollution accounts for approximately 12% of the global burden of disease (Zhou et al., 2019). A growing number of epidemiological studies have demonstrated that UFPM is closely associated with morbidity and mortality due to both acute and chronic cardiovascular diseases (Ruckerl et al., 2007). Chen et al. reported that UFPM could trigger the onset of nonfatal myocardial infarction at a sub-daily timescale (Chen et al., 2020). Short-term acute exposure or long-term exposure to fine PM can cause acute cardiovascular events such as myocardial ischemia (MI), ischemic stroke, and congestive heart failure (Wei et al., 2019), which are favored by the smaller particles (Araujo and Nel, 2009). Consequently, UFPM can exert significant adverse effects on patients with cardiovascular disease, resulting in a large number of deaths.

Several mechanistic pathways are responsible for the cardiovascular effects of acute and chronic exposures to UFPM (Chin, 2015; Hadei and Naddafi, 2020). The translocation of pollutants may increase blood pressure, cause endothelial injury/dysfunction, and induce systemic oxidative stress (OS) (Lee et al., 2014) and inflammation (Tsai et al., 2012), which can cause thrombosis, coagulation (Chiarella et al., 2014), and arterial vasoconstriction (Rao et al., 2014) and decrease the heart rate variability, leading to MI events. Divergence in the toxicological mechanisms of PM could be attributed to the different origins of air particles with distinct chemical components that can trigger various pathways (Ronkko et al., 2018). Although different signaling pathways may be involved, systemic OS plays a major role in the cardiovascular effects of PM pollutants (Li R. et al., 2015; Miller, 2020).

Numerous treatment measures to alleviate the risk of PM exposure have been adapted and evaluated, including the use of masks or air purifiers and antioxidants (Zhong et al., 2017; Bolcas et al., 2019). In addition, extracts of traditional medicinal plants and natural products have been used to attenuate or prevent PM-induced injury in laboratory animals (Lee et al., 2019; Sanjeewa et al., 2020). Zhang reported that walnut protein isolates exerted protective effects in a PM-induced acute lung injury mouse model. The walnut protein isolates inhibited myeloperoxidase (MPO), nitric oxide (NO), interleukin-1β (IL-1β), and interleukin-6 (IL-6) in bronchoalveolar lavage fluid. In addition, pro-inflammatory cytokine production and acyl carrier protein levels were decreased by the walnut extract (Zhang et al., 2019). Tanshinone IIA effectively reduced PM_2.5_ damage to EA.hy926 cells by inhibiting the p38/MAPK pathway (Jia et al., 2012). However, the therapeutic or preventive effects of these agents on MI events following exposure to UFPM have not been reported.

Shengmai formula (SMF) is composed of Ginseng radix et Rhizoma Rubra (*Panax ginseng* C. A. Mey.), Ophiopogonis Radix [*Ophiopogon japonicus* (Thunb.) Ker Gawl.], and Schisandrae Chinensis Fructus [*Schisandra chinensis* (Turcz.) Barll.]. SMF has long been used clinically for the treatment of heart failure and has outstanding curative effects on cardiovascular diseases owing to its free radical scavenging activity (Li F. et al., 2015). In addition, SMF increases superoxide dismutase (SOD) activity and reduces inflammatory and OS activities to protect the myocardium and strengthen the heart (Zhu et al., 2019). SMF is a strong natural antioxidant used in traditional Chinese medicine and has a 2000-year history of use. SMF has cardioprotective properties and strong antioxidant, anti-atherogenic, and immune-modulating effects (Ichikawa et al., 2003).

In the present study, the protective effects of SMF on myocardial injury after exposure to UFPM were tested in rat and H9C2 cell models. The aims of this study were to determine whether SMF could inhibit UFPM-induced OS responses in rats with MI and improve the recovery of myocardial tissue damaged following exposure to UFPM + MI. In addition, the potential mechanism involved in the process was examined. Network pharmacology, RT-qPCR, western blot analysis, and inhibitors were used to investigate the pharmacological effects of SMF on myocardial injury and identify potential therapeutic targets and pathways.

## Materials and Methods

### Animals and Reagents

Healthy adult male Sprague Dawley rats weighing 200 ± 20 g were purchased from the China Institute of Food and Drug Testing. All rats were raised in light-controlled and air-conditioned (23 ± 2°C) rooms and had free access to food and water. This study was approved by the Animal Ethics Committee of the Institute of Chinese Materia Medica, China Academy of Chinese Medical Sciences. All reagents were purchased from Sigma-Aldrich (Missouri, United States) unless otherwise stated. UFPM (NIST® SRM® 1650b) was obtained from MilliporeSigma Corporate Offices (CAS Number 1333-86-4), and chemical characterization of UFPM can be found at https://www-s.nist.gov/srmors/certificates/1650b.pdf.

### Quality Control and Preparation of Shengmai Formula

SMF was purchased from Beijing Tongrentang Co., Ltd. (batch number, 16262159). Beijing Tongrentang Co. is a Chinese pharmaceutical company founded in 1669 and is the largest producer of traditional Chinese medicine. The drug is a multi-herbal preparation prepared according to the Pharmacopoeia of the People’s Republic of China (Chinese Pharmacopoeia Commission, 2010). High-performance liquid chromatography-mass spectrometry (HPLC-MS) was used to identify the main chemical components of SMF. The active ingredients are shown in Supplementary Figure S1; Supplementary Table S1. The concentration of two major compounds in SMF, schisandrin (C_24_H_32_O_7_) and ruscogenin (C_27_H_42_O_4_), were 0.022 mg/mg and 0.132 mg/mg, respectively, as determined using HPLC and ultraviolet-visible (UV-Vis) spectrometry. The chromatogram for schisandrin is shown in Supplementary Figure S1.

For the preparation of SMF, Ginseng radix et Rhizoma Rubra, Ophiopogonis Radix, and Schisandrae Chinensis Fructus were mixed at a ratio of 1:2:1 (w/w). These three ingredients were pulverized to a coarse powder and macerated in 65% ethanol for 24 h. Approximately 4,500 ml of percolate was collected and concentrated to approximately 250 ml under vacuum. After cooling, the solution was diluted with 400 ml of water and filtered. Next, 300 ml of 60% syrup and a quantity of preservative was added, the pH was adjusted to the specified range, and the volume was adjusted to 1000 ml. After stirring thoroughly, the solution was allowed to stand, filtered, packed, and sterilized. For detailed information on the three herbal ingredients in SMF, see Supplementary Table S2. The structures of active compounds in SMF are shown in Supplementary Figure S2.

### SMF Administration, UFPM Exposure, and Myocardial Infarction Model

The rats were anesthetized with chloral hydrate (60 mg/kg, i.p.), and the specified dose of SMF or saline was given by oral administration daily for 7 days. Twenty-four hours after the final SMF administration, the animals were intubated with a tracheal tube through which 2.0 mg of UFPM suspended in 0.3 ml of saline was instilled (purchased from National Institute of Standards and Technology, Maryland, United States). MI was induced by ligating the left anterior descending coronary artery, as previously described (Wu et al., 2011; Li et al., 2012). The coronary artery was not ligated in the sham group. Specifically, the MI model with UFPM exposure was constructed by ligating the left anterior descending coronary artery, followed by 24 h UFPM exposure. Subsequently, the rats were killed after 24 h of ischemia.

### Animal Experimental Design

**FIGURE 1:**
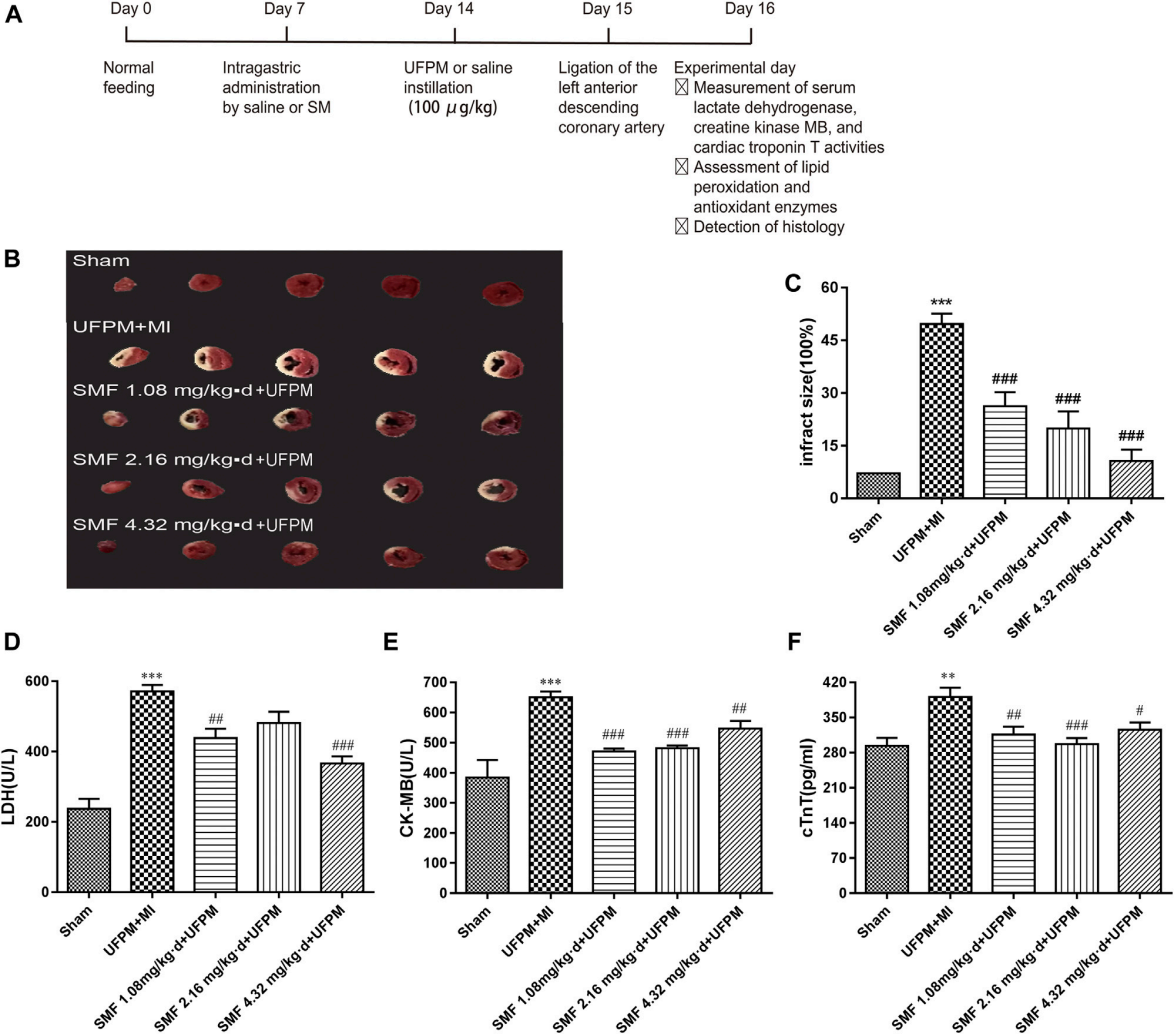
SMF pretreatment protected against MI in UFPM-exposed rats with MI. **(A)** Treatments and endpoints following intratracheal instillation of saline or ultrafine particulate matter (UFPM) with or without repeated Shengmai formula (SMF) pretreatment (administered by oral gavage) in rats with myocardial ischemia (MI). **(B)** Isolated rat hearts taken after LAD ligation followed by 24 h of ischemia were sectioned and stained with TTC. **(C)** Infarct size quantification by TTC staining. Infarct sizes were quantified and expressed as a percentage of the total left ventricle area. Ratios were obtained in six rats per group (means ± SEM). Levels of **(D)** LDH, **(E)** CK-MB, and **(F)** cTnT in rat sera. All values are expressed as mean ± SD, *n* = 6 per group. LAD, left anterior descending; TTC, triphenyltetrazolium chloride. *p < 0.05, **p < 0.01 vs. sham. ^#^ *p* < 0.05, ^##^ *p* < 0.01 vs. UFPM + MI.

The rats were randomly divided into five groups (*n* = 10 each) as follows: sham group; UFPM + MI group, treated with saline for 7 days before administration of UFPM followed by 24 h of ischemia; SMF (1.08 mg/kg⋅d, orally, 7 days) + UFPM + MI group, treated with SMF for 7 days before UFPM instillation, followed by 24 h of ischemia; SMF (2.16 mg/kg⋅d, orally, 7 days) + UFPM + MI group, treated with SMF for 7 days before UFPM instillation, followed by 24 h of ischemia; and SMF (4.32 mg/kg⋅d, orally, 7 days) + UFPM + MI group, treated with SMF for 7 days before UFPM instillation, followed by 24 h of ischemia (Figure 1A). To measure the effect of SMF on cardiac function from the pathological and blood indexes of heart tissue, lactate dehydrogenase (LDH), creatine kinase myocardial band (CK-MB), cTnT levels, triphenyl tetrazolium chloride (TTC) staining and hematoxylin and eosin (H&E) assays were performed. To further clarify which biological processes are affected by SMF, we tested the oxidative stress response based on previous reports. Generally, mitochondrial damage can lead to oxidative stress reaction. Therefore, we evaluated mitochondrial function-related indicators [mitochondrial membrane potential (MMP), Seahorse and mitochondrial ultrastructure], levels of myocardial malondialdehyde (MDA), NADPH oxidase (NOX), heme oxygenase-1 (HO-1), catalase (CAT), total SOD (T-SOD), and phospholipid hydroperoxide glutathione peroxidase (GSH-Px) and mRNA levels of *SOD1*, *CAT*, *GSH-Px*, and *NOX* in myocardial tissues. Furthermore, we sought to determine which pathway or molecule mediates the changes in oxidative stress.

### Quantification of Infarct Size (Rats)

The infarct size was determined as described previously (Gao et al., 2011). Briefly, the heart was promptly removed after euthanasia and stored for 8 min at −80°C. Then, 2-mm-thick sections were cut, stained with 1% 2,3,5-triphenyl tetrazolium chloride (TTC) in phosphate buffer (pH 7.4) for 20 min at 37°C, and fixed overnight in 10% formalin. Normal myocardium was stained red by TTC, whereas infarcted myocardium was pale in color due to cell membrane damage. Images of the slices were acquired, and the myocardial infarct area was calculated as a percentage of the total area using Image-Pro Plus 6.0.

### Measurement of Serum Lactate Dehydrogenase, Creatine Kinase MB, and Cardiac Troponin T Activities

LDH and CK-MB activities in serum were assessed using commercial kits (Nanjing Jiancheng Bioengineering Institute, Nanjing, China) according to the manufacturer’s protocols, as described previously (Kim et al., 2017). LDH and CK-MB activities were expressed in U/L. Blood samples were obtained 24 h after MI, and serum cTnT levels were measured (pg/ml) using a commercial assay kit (ab246529, Cambridge MA, United States) according to the manufacturer’s instructions, as described previously (Hortmann et al., 2017).

### Assessment of Lipid Peroxidation and Antioxidant Enzymes

MD, NOX, HO-1, CAT, T-SOD, and GSH-Px levels in the heart and serum were measured using commercial kits (Nanjing Jiancheng Bioengineering Institute, Nanjing, China).

### Histology

Twenty-four hours after MI induction, the hearts were removed, fixed in 4% paraformaldehyde, and embedded in paraffin. The paraffin-embedded tissues were sliced into 5-mm sections and stained with hematoxylin and eosin (H&E). An optical microscope (Olympus, Japan) was used to observe pathological changes in the tissues. To determine the myocardial ultrastructure, the myocardium was fixed in 3% glutaraldehyde, followed by fixation in 1% osmium tetroxide and dehydration in ethanol. Epoxy resin was used to embed the tissues, followed by the use of a hardener, accelerator, and growth agent. Subsequently, 70-nm-thick sections were cut using an ultra-microtome and stained with a solution containing uranyl acetate and lead citrate. Changes in the myocardial ultrastructure were observed using a JEM-1200EX transmission electron microscope (TEM, JEM 1200EX II, Jeol, Japan).

### Network Pharmacology-Based Analysis

An integrated pharmacology network-computing platform for traditional Chinese medicine (TCMIP V2.0, http://www.Tcmip.cn,updated on September, 2019) (Xu et al., 2019) and the SymMap databases (https://www.symmap.org/) (Wu et al., 2019) were used to collect the SMF compounds. TCMIP V2.0, SymMap, and Swiss Target Prediction (http://www.swisstargetprediction.ch/index.php) databases were used to predict the targets of active ingredients. Disease-associated target prediction was performed using DisGeNET (http://www.disgenet.org/web/DisGeNET/), Therapeutic Target Database (TTD), and Human Phenotype Ontology (HPO) databases and published literature. The potential targets of SMF were predicted using the DrugBank database (http://www.drugbank.ca/, version: 3.0). The relevant protein–protein interaction network was extracted from the Human Protein Reference Database and String databases. This information along with information about the herbs, ingredient-related proteins, and disease-related proteins were entered into a bioinformatics software, Cytoscape (version 3.7.1), to construct a complete network for SMF. The Kyoto Encyclopedia of Genes and Genomes (KEGG) pathway database was used to analyze the disease-related targets of SMF and toxicity-related pathways of UFPM.

### Experimental Validation

#### Oxidative Stress-Related Gene Expression Analysis

Mitochondria of the heart tissue were homogenized and lyzed with Trizol reagent (Life Technologies) to extract total RNA after 24 h of MI. The extracted RNA samples were purified using the spin column-based RNeasy Mini Kit (Qiagen, Hilden, Germany). The quality and concentration of RNA were measured using a NanoDrop® ND-1000 spectrophotometer (Thermo Fisher Scientific, Massachusetts, United States). The A260/280 values of all the RNA samples were 1.8–2.1. cDNA was transcribed using the RT^2^ First Strand Kit (Qiagen, Hilden, Germany). The RT^2^ Profiler PCR array (Qiagen, 48-well format, catalog no. PARN-065ZA) was used to analyze gene expression using a Stratagene Mx3005P real-time (RT) quantitative polymerase chain reaction (qPCR) system (Agilent Technologies).

#### RT-qPCR Analysis

RT-qPCR assays for *Nrf2*, *HO-1*, *CAT*, *T-SOD*, *Gpx1*, *NQO1*, *NoX4*, and *GAPDH* (housekeeping gene) were performed for further validation (see Supplementary Table S3 for primers). RNA was isolated and cDNA was synthesized using the High Capacity cDNA Reverse Transcription Kit (Invitrogen Life Technologies). Gene expression levels were determined by RT-qPCR using the SYBR Green PCR Master Mix (Qiagen, Hilden, Germany) and normalized to the level of the housing gene *GAPDH*. Gene expression levels in the SMF-treated group were expressed as fold-changes when compared with gene expression levels in the UFPM-exposed MI rats using the 2^−∆∆CT^ method. Similarly, the gene expression levels in the UFPM-exposed MI rats were determined by calculating the fold-change when compared with the sham control rats.

#### Cell Culture and Treatment

Rat myoblast cells (H9C2) were purchased from American Type Culture Collection and cultured in Dulbecco’s modified Eagle’s medium supplemented with 10% fetal bovine serum, 100 U/mL penicillin, and 100 μg/ml streptomycin (Gibco, Madison, United States) under sterile conditions. The cells were incubated with 5% CO_2_ at 37°C. For the cell viability assay, H9C2 cells were exposed to UFPM (25, 50, 100, and 200 mg/ml) for 24 h or SMF (0.28, 0.56, 1.12, and 2.24 mg/ml) for 24 h. Cell Counting Kit 8 (WST-8/CCK8) (ab228554, Abcam) was used for analysis. To analyze the mechanism of SMF, H9C2 cells pre-incubated with either medium or LY294002 (50 μM)/SB 203580 (10 mM) for 4 h were then cultured with medium or SMF for 24 h followed by culture with or without UFPM (50 mg/ml) for 6 h. The lysates from H9C2 were resolved on 10% SDS-PAGE, transferred onto membranes, and blotted with specific antibodies. The supernatants were collected for analysis of OS biomarkers (T-SOD, CAT, GSH-PX, and NOX) using ELISA assays.

#### Seahorse Assay

The XFe96 Extracellular Flux Analyzer (Seahorse Bioscience) was used to measure oxygen consumption rates (OCRs) and extracellular acidification rates. The cells were diluted to 2× the final optical density, and 90 μl of cell culture was added to XF Cell Culture Microplates pre-coated with poly-D-lysine. The cells were centrifuged for 10 min at 1,400 × *g* in a Heraeus Multifuge ×1R (M-20 rotor) to attach them to the pre-coated plates. After centrifugation, 90 μl of fresh media was added to each well. To achieve uniform cell seeding, the initial OCR was measured for two cycles (7 min) before UFPM exposure. The maximal OCR that can be read on the analyzer is approximately 700–800 pmol/min; above this rate, the consumption rate exceeds the replenishment of the system and the curves show a false declination in OCR, which was excluded from the graphical presentation.

#### Western Blotting

Proteins were extracted from frozen heart tissues. The bicinchoninic acid protein assay was used to determine the protein concentration using bovine serum albumin as a standard (Pierce Rockford, IL). Equal amounts of proteins were loaded on SDS-PAGE gels; after separation, the proteins were transferred to a polyvinylidene difluoride membrane. The membranes were incubated with anti-MAPK p38 (CST, #8690S), p-MAPK p38 (CST, #4511S), rat anti-AKT (ab8805, Abcam), p-AKT (ab81283, Abcam), rabbit anti-Nrf2 (1:1000, 16396-1-AP, Proteintech), rabbit anti-PCNA (1:1000, 10205-2-AP, Proteintech), rabbit anti-HO-1 (1:1000, #82206S, CST), and rabbit antibody to β-Actin (1:2500, #8457, CST) antibodies. The bands were visualized by enhanced chemiluminescence and scanned using FLUORCHEM™ E (ProteinSimple, United States). The densitometric results were quantified using ImageJ software. The results from western blotting were normalized to the control group.

MAPK inhibitor (SB203580) was purchased from Merck-Millipore (Billerica, MA, United States), and PI3K inhibitor (LY294002, ab120243) was purchased from Abcam.

### Statistical Analysis

Data were expressed as mean ± standard error of the mean. One-way analysis of variance was used to analyze the differences among the experimental groups. Dunnett’s multiple comparison test was used for inter-group comparisons. A *p*-value of less than 0.05 was considered statistically significant.

## Results

### Effects of UFPM Exposure, MI, and SMF on Survival Rates

Survival rates after UFPM exposure and 24 h of MI are shown in Table 1. The survival rate was 45% after UFPM exposure + MI and decreased compared with that in the sham group. Pretreatment with SMF (UFMI + MI + SMF groups) improved survival compared with that in the UFPM + MI group. Survival rates after 1.86, 2.16, and 4.32 mg/kg⋅d SMF pretreatment were 66.7, 86.7, and 73.3%, respectively.

**TABLE 1:** Survival rates (%) after SMF pretreatment in UFPM-exposed rats with MI.

Groups^a^	SMF dose (mg/kg⋅d)	Survival rate (%)
Sham	—	66.7
UFPM + MI	—	45.0
UFPM + MI + SMF	1.08	66.7
	2.16	86.7
	4.32	73.3

^a^ *n* = 15 animals per group. SMF, Shengmai formula; UFPM, ultrafine particulate matter; MI, myocardial ischemia.

### MI Size Quantification

As shown in Figures 1B,C, a significant difference in myocardial infarct size was observed between the experimental and control groups. The myocardial infarct size in the UFPM-exposed MI group (49.69 ± 7.167) was significantly larger than that in the sham group (10.22 ± 2.739). Moreover, the infarct sizes were smaller in the SMF-treated groups than in the UFPM-exposed MI group (26.29 ± 9.684, 19.91 ± 11.92, and 10.56 ± 8.197 in the 1.86, 2.16, and 4.32 mg/kg⋅d SMF pretreatment groups, respectively). A typical example of a myocardial infarct is shown in Figure 1B.

### Serum Markers of Myocardial Damage

Serum markers of myocardial damage are shown in. LDH levels significantly increased in the UFPM-exposed MI group compared with that in the sham group. However, SMF pretreatment significantly restored the LDH values to close to sham levels (Figure 1D). Furthermore, the CK-MB (212.3 U/L) and cTnT (97.7 pg/ml) levels in the UFPM-exposed MI group were significantly higher than those in the sham group (Figures 1E,F). These values were significantly improved by pretreatment with SMF in the UFPM-exposed MI group. These results indicate that SMF attenuated cardiac injury induced by UFPM exposure and MI.

### Histopathological Assessment of Heart Tissues

**FIGURE 2:**
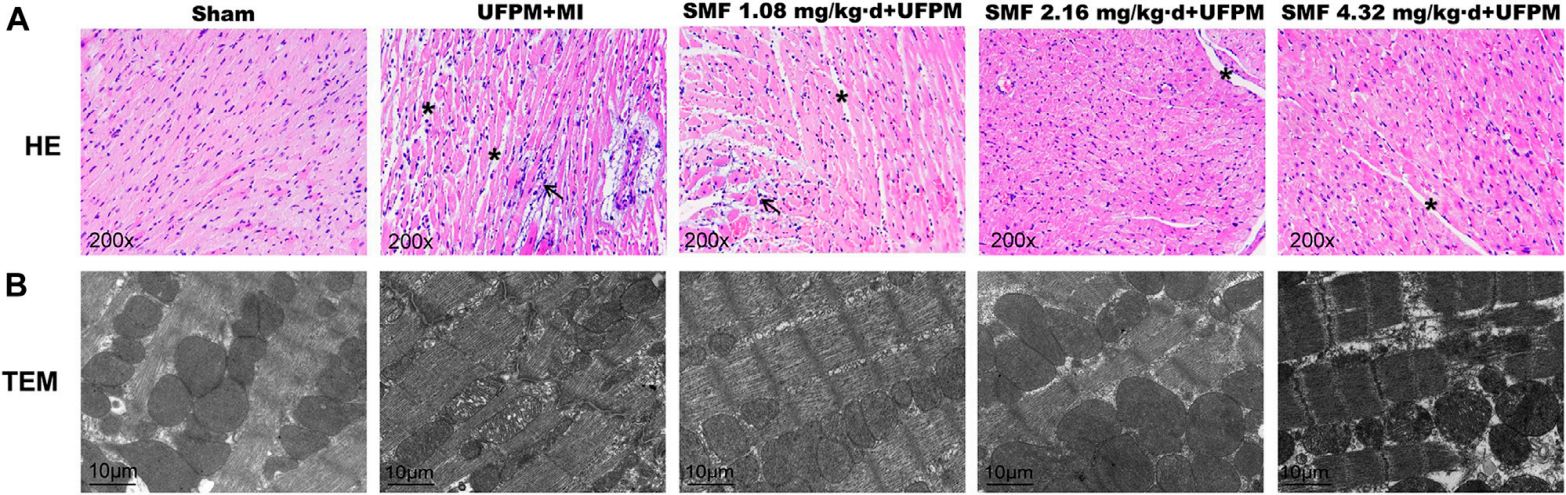
Protective effects of SMF on toxic changes in myocardial tissues in acute UFPM-exposed rats with MI, as shown by hematoxylin and eosin (H&E) staining and transmission electron microscopy (TEM). Representative images of H&E staining (**A**, ×200) and TEM (**B**, bar = 10 μm) in myocardial tissues from the sham or MI groups pretreated with different SMF concentrations before UFPM exposure. MI, myocardial ischemia; SMF, Shengmai formula; UFPM, ultrafine particulate matter.

To evaluate the effects of SMF on cardiac morphology, myocardial sections were stained with H&E (Figure 2A). The myocardial muscle fibers in the sham group were regularly arranged, and no edema was observed between the cells. In contrast, the tissues in the UFPM-exposed MI group exhibited moderate vascular edema, myocardial fiber edema, connective tissue hyperplasia, and enlarged myocardial fibers with inflammatory cell infiltration. These morphological signs of myocardial injury were attenuated in the SMF pretreated groups. Specifically, heart tissues in the low-dose SMF pretreatment group showed local hemorrhage, myocardial fiber edema, red blood cells between the myocardial fibers, and enlarged myocardial fibers with inflammatory cell infiltration. Myocardial tissues in the middle- and high-dose SMF pretreatment groups showed decreased numbers of red blood cells, reduced intracellular gaps between myocardial fibers, and reduced infiltration of inflammatory cells compared with myocardial tissues in the UFPM-exposed MI group.

The myocardial ultrastructure in rats in the sham group was generally normal (Figure 2B). The myocardial cells and nuclear membranes were intact, no edema was detected, the myofibril structure was neatly arranged, the mitochondrial structure was intact, the ridges were dense, and no swelling or vacuole-like changes were observed. The myocardial ultrastructure in rats in the UFPM + MI group demonstrated abnormal changes, including edema in some myocardial cells, blurred light and dark bands of muscle segments, broken myofilaments, swollen mitochondria, dissolved ridges, and vacuole-like changes. In the low-dose SMF pretreatment group, the size and number of lesions were reduced. Specifically, myofibrils were neatly arranged, the sarcomere structure was clear, the mitochondria were slightly swollen, and vacuole-like changes were observed in the low-dose SMF group. In the middle-dose SMF group, the size and number of myocardial lesions were also reduced compared with that in the UFPM + MI group, with fewer mitochondrial lesions, neatly arranged myofibrils, and clearly-structured sarcomeres. Moreover, the mitochondrial structure was found to be swollen occasionally and the palate was regular and dense.

### Biomarkers of OS in Heart Tissue

**FIGURE 3:**
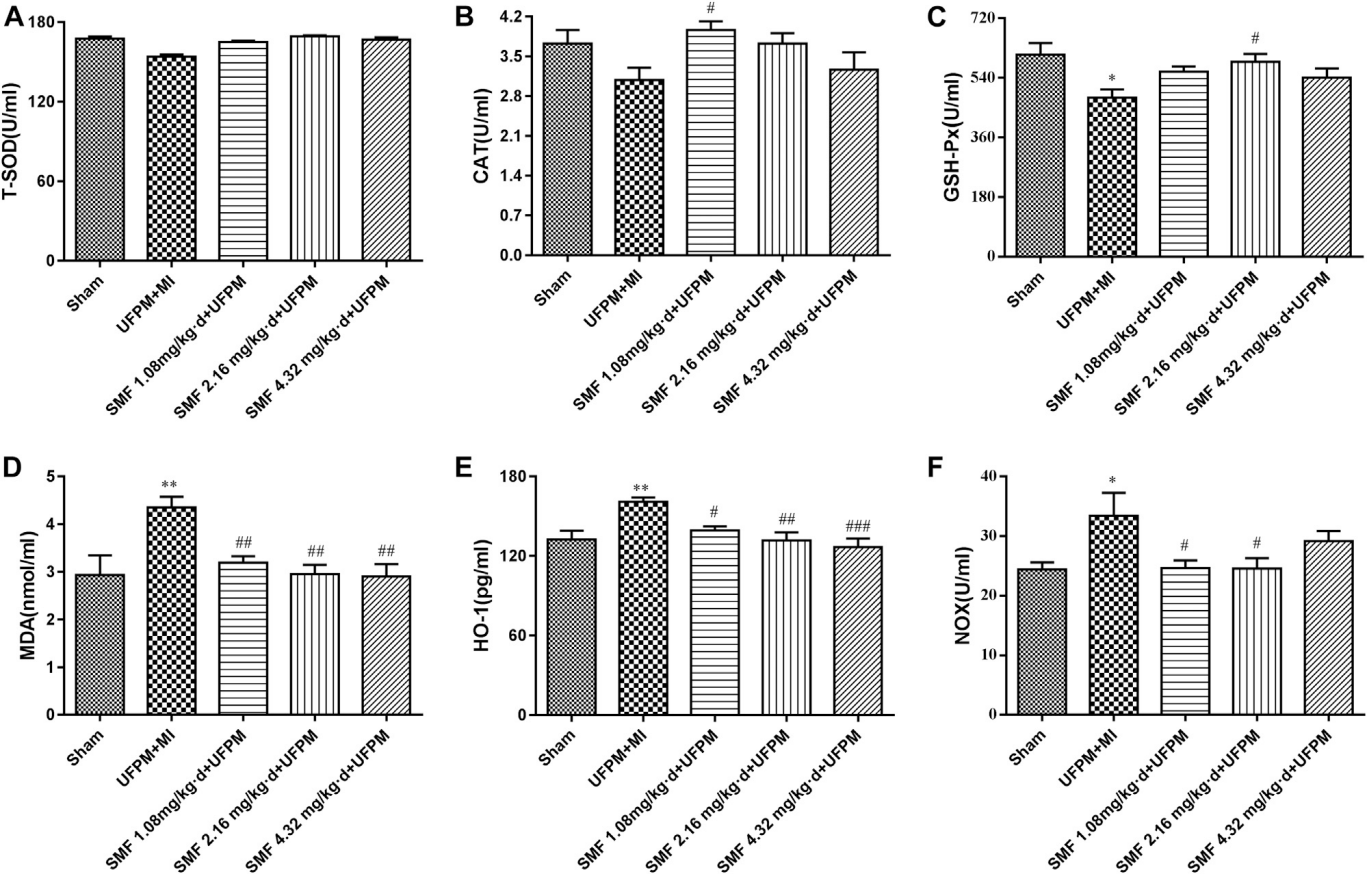
Effects of SMF on UFPM-induced oxidative stress in rats with MI. **(A)** T-SOD (total superoxide dismutase), **(B)** CAT (catalase), and **(C)** glutathione peroxidase (GSH-Px) activity were measured in serum from the five experimental groups.**(D)** Malondialdehyde (MDA), **(E)** Heme oxygenase-1 (HO-1), and **(F)** NADPH oxidase (NOX) were analyzed. Data are represented as mean ± SEM. * and **, significant difference from sham group at *p* < 0.05 and *p* < 0.01; ^#, ##^ and ^###^, significant differences from the UFPM group at *p* < 0.05, *p* < 0.01, and *p* < 0.001, respectively. MI, myocardial ischemia; SMF, Shengmai formula; UFPM, ultrafine particulate matter.

Significant reductions in the levels of T-SOD, CAT, and GSH-Px (lowest levels reaching 13.5, 0.649, and 130 U/mL, respectively) were observed in the UFPM + MI rats compared with that in the sham group (Figures 3A–C). However, SMF pretreatment significantly restored the levels of antioxidant enzymes to those in the sham group. The HO-1, NOX, and MDA levels were increased significantly in the UFPM + MI rats compared with those in the sham group (*p* < 0.05), and these changes were attenuated in the SMF pretreatment groups (*p* < 0.05; 26.08 ± 5.0 vs. 33.4 ± 10.8 for NOX, 3.01 ± 0.6 vs. 4.36 ± 0.62 for MDA), as shown in Figures 3D–F. These results indicate that pretreatment with SMF improves the OS response to UFPM + MI.

### Network Pharmacology Analysis

To identify the molecular mechanism by which SMF attenuates myocardial damage in response to UFPM and MI, a UFPM-cardiovascular-drug target-pathway network analysis was performed based on the related proteins and their signaling pathways. Putative targets of SMF, composed of Ginseng radix et Rhizoma Rubra, Ophiopogonis Radix, and Schisandrae Chinensis Fructus, were predicted using the DrugBank database. A total of 475 putative targets for SMF were identified; 376 genes were predicted as putative targets after removing the duplicates (Supplementary Table S4). The cardiovascular-related genes were carefully collected from DisGeNET (3,240 genes), HPO (1,018 genes), and TTD (2,398 genes), and previously published research articles (1,347 genes) are listed in Supplementary Table S5. Subsequently, 1,343 genes were collected after removing the duplicates. A network was constructed, including the 376 predicted genes and 1,343 cardiovascular-related genes. The cutoff value was set at 0.626, and a total of 1,214 nodes and 6,370 pairs of interactions were included in the network.

**FIGURE 4:**
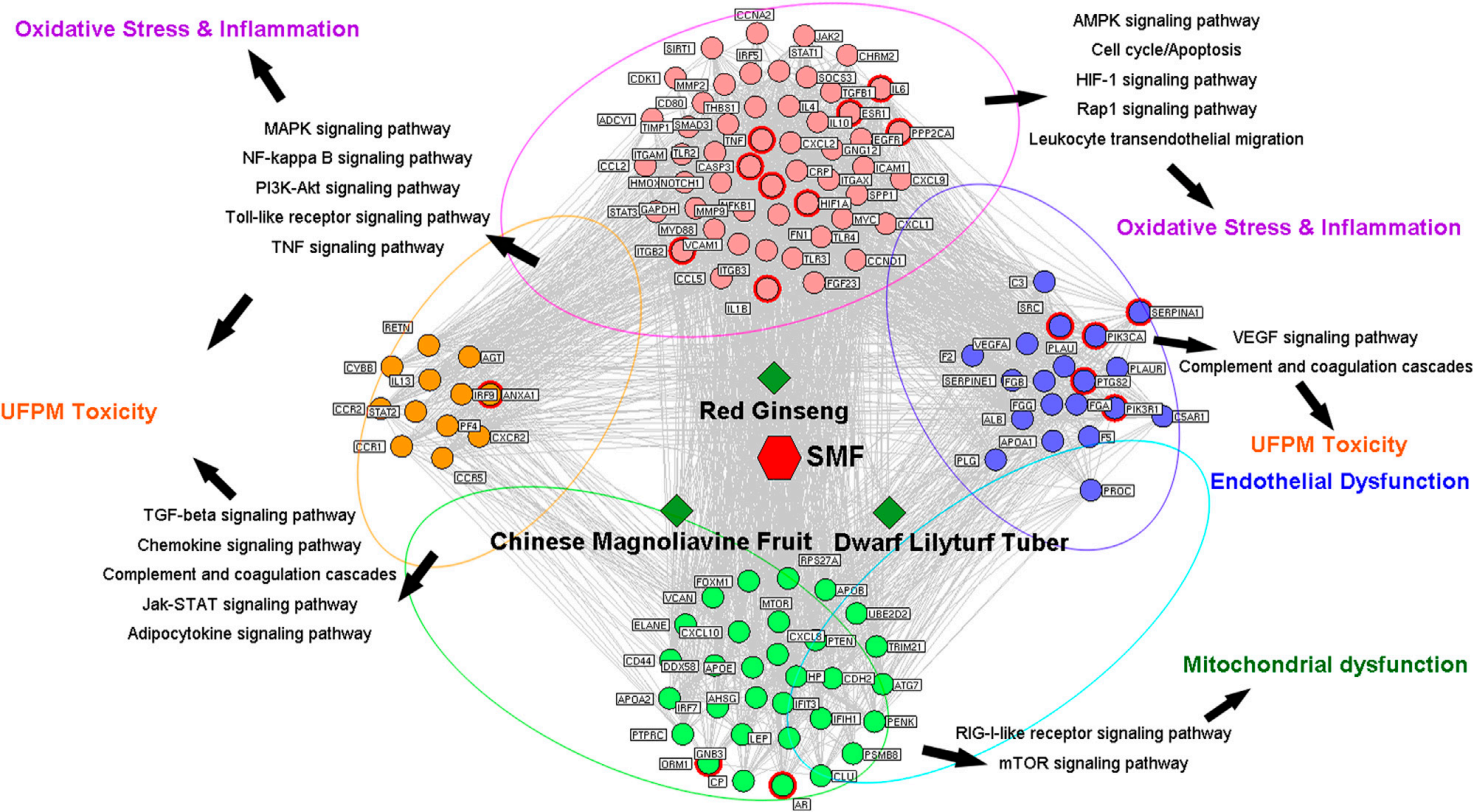
UFPM, Cardiovascular disease-drug target-pathway network. The red node at the center of the figure corresponds to the Shengmai formula and the surrounding green diamond-shaped nodes are the three Chinese herbal medicines (Ginseng radix et Rhizoma Rubra, Ophiopogonis Radix, and Schisandrae Chinensis Fructus). The round nodes represent the key network targets in the “disease gene-drug target interaction network,” where the red circles (the marked target genes) are candidates for the Shengmai formula, and the others are disease-related genes. The edges represent interactions among cardiovascular disease genes, herbs, and putative targets for the treatment of cardiovascular disease. The different color ovals represent the main pathways from the enrichment analysis of major targets in the “disease gene-drug target interaction network” and the previously described toxicity pathways of UFPM.

The hubs in a network have excessively high levels of node degree and are likely to be major genes. Based on previous studies, we defined a node as a hub if the node degree was more than twice the median degree of all the nodes in the network (Li S. et al., 2007). Consequently, 354 nodes were identified as hubs, and an interaction network comprising these nodes was constructed (Supplementary Table S6). By calculating the topological characteristic values of these nodes based on the hub interaction network (“Degree,” “Node betweenness,” and “Closeness”), the major nodes and key network targets of SMF action were successfully screened, and 116 key network targets were obtained (Supplementary Table S7). A pathway enrichment analysis of the above network targets was performed based on the KEGG pathway by combining the UFPM toxicological pathways in cardiovascular diseases (CVDs) and the literature (Figure 4; Supplementary Table S8). Among the enriched pathways, the two largest functional modules were OS (PI3K/AKT, MAPK, and AMPK signaling pathways) and inflammation pathways (p53, chemokine, and NOD-like receptor signaling pathways and cytokine–cytokine receptor interactions). The other modules were involved in mitochondrial dysfunction and endothelial dysfunction. For SMF, IL-6, ESR1, PPP2CA, TNF, CASP3, NFKB1, ITGB2, IL-1β, HIF1A, SERPINE1, PIK3CA, PLAU, PTGS2, PIK3R1, and GNB3 were identified as major targets (red circle in Figure 4) that play essential roles in OS and inflammation and were regarded as the key markers of SMF treatment in CVD. Notably, the putative targets of TNF, CASP3, NFKB1, and IL-1β were involved in the p38 MAPK signaling pathway and were strongly associated with the pathological, toxicological, and pharmacological mechanisms of CVD, UFPM, and SMF, respectively. The p38 MAPK signaling pathway can be stimulated by numerous stress signals, including OS, leading to the production of antioxidants. Based on the integrated UFPM-cardiovascular-drug target-pathway network, we propose that the antioxidant effects of SMF in rats subjected to UFPM exposure and MI are associated with key targets (TNF, CASP3, NFKB1, and IL-1β) regulating the OS response.

Furthermore, to gain the main chemical composition, we screened candidate compounds and predicted the gene targets of SMF (Supplementary Table S9), and an ingredient-target network of SMF was established. After eliminating all duplicate compounds and overlapping targets, SMF yielded 56 candidate compounds and a total of 290 targets were identified (Supplementary Figure S3). Specifically, the network included 155 nodes and 268 ingredient-target interactions. In the network, we found that IL-1β, TNF, CASP3, IL-4, FN1 and CCND1, which were involved in p38 MAPK/PI3K/AKT pathway, were targets of Kaempferol, Ginsenoside Rd, Ginsenoside Rf, Ginsenoside Rg1, Ginsenoside Rh2, 20-Glucosylginsenoside Rf, D-Limonene, Geraniol and Citral-B, as highlighted in Supplementary Figure S3. Additionally, by HPLC-MS assay, we identify the main chemical components of SMF, including Ginsenoside Rf, Ginsenoside Rg1, Ginsenoside Rh2, which were described above. Thus, we speculate that Ginsenoside Rf, Ginsenoside Rg1, Ginsenoside Rh2 could be the main chemical composition by which SMF play its protection roles *via* PI3K/AKT/p38 MAPK/Nrf2 pathway.

### Experimental Validation

#### Evaluation of H9C2 Cell Viability After UFPM Exposure and SMF Treatment

H9C2 cells were exposed to various concentrations of UFPM, and alterations in cell viability were observed using the CCK8 assay. A dose-dependent decrease in cell survival was observed following UFPM exposure (Supplementary Figure S4A). Cells exposed to high doses of UFPM (50, 100, and 200 mg/ml) demonstrated significant reductions in cell viability (*p* < 0.05). The cell survival rate after 100 μg/ml UFPM exposure was approximately 57%. This group was selected for subsequent *in vitro* studies.

To examine the functional role of SMF, H9C2 cells were pretreated with 0.28, 0.56, 1.12, and 2.24 mg/ml SMF before UFPM exposure and cell viability was measured (Supplementary Figure S4B). Pretreatment with SFM resulted in significantly improved viability in UFPM-treated cells (concentrations 0.28, 0.56, and 1.12 mg/ml) compared with the vehicle (*p* < 0.05).

#### SMF Suppresses UFPM-Induced ROS Production in H9C2 Cells

UFPM induces ROS generation to promote OS. Therefore, we examined whether SMF could decrease ROS generation and the expression levels of ROS-dependent genes. First, we detected ROS by H2DCF-DA staining. Supplementary Figure S4C shows that the ROS levels in the H9C2 cells after UFPM exposure were significantly higher than those in the control cells. This effect was attenuated by SMF.

#### SMF Attenuated UFPM-Induced Mitochondrial Damage in H9C2 Cells

**FIGURE 5:**
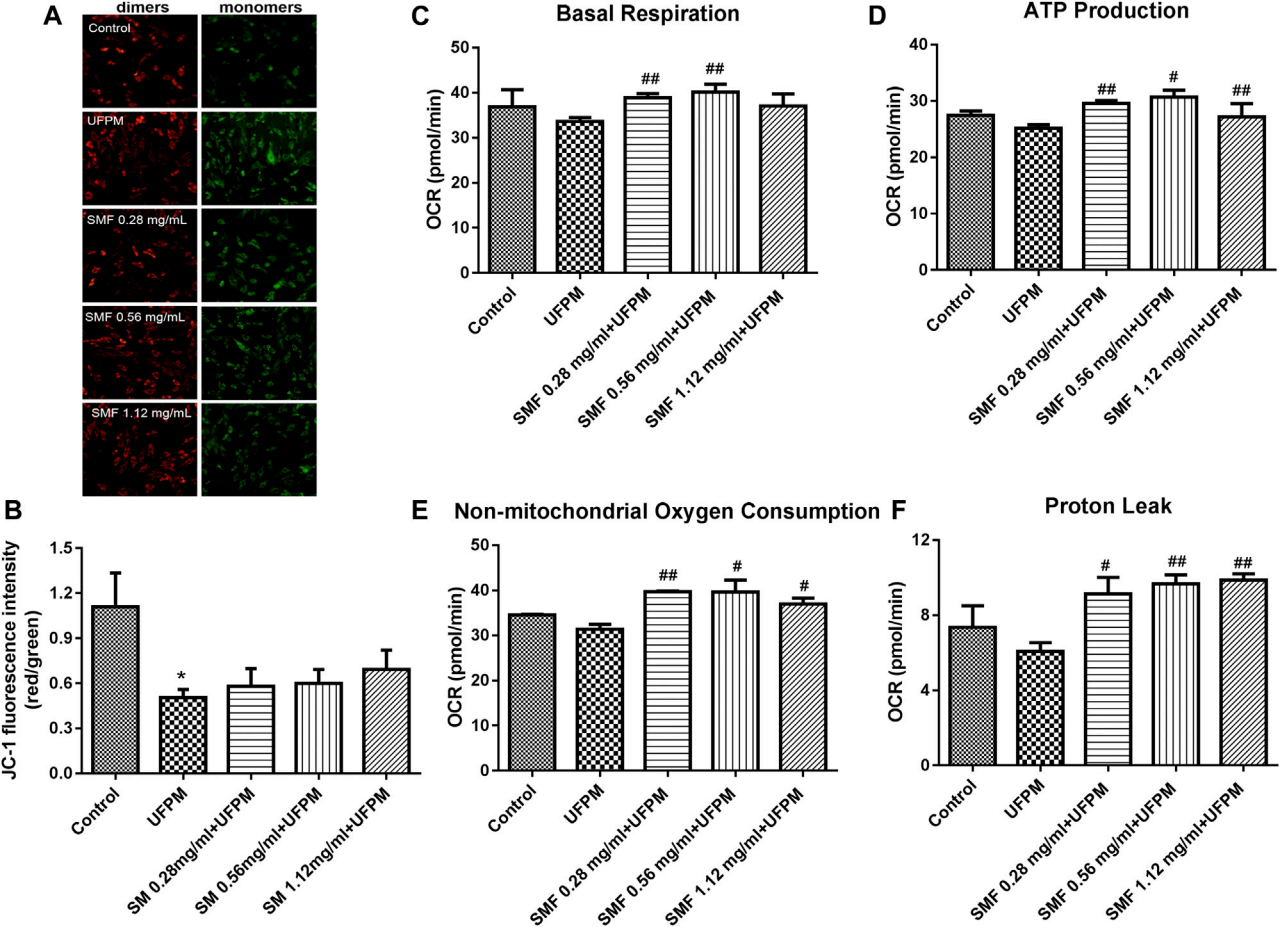
SMF attenuated UFPM-induced mitochondrial damage in H9C2 cells. **(A)** Effects of the indicated concentrations of SMF on mitochondrial membrane potential in H9C2 cells. Representative images are shown. **(B)** The optical density ratio of the red and green fluorescence is shown. *p < 0.05, compared to the control group. **(C–F)** SMF improves *in vitro* UFPM-induced mitochondrial activity. Basal respiration, ATP production, non-mitochondrial respiration, and proton leak of cells exposed to UFPM with or without SMF pretreatment. Oxygen consumption rate (OCR) was measured with a Seahorse metabolic analyzer. The data are presented as mean ± standard deviation (*p < 0.05, **p < 0.01 compared to the control group; ^#^ *p* < 0.05, ^##^ *p* < 0.01, ^###^ *p* < 0.001 compared to the UFPM group). MI, myocardial ischemia; SMF, Shengmai formula; UFPM, ultrafine particulate matter.

Previous studies showed that UFPM-induced OS promoted changes in the MMP. As shown in Figures 5A,B, JC-1 exhibited a monomeric form in UFPM-exposed cells and Δψm significantly decreased (*p* < 0.05). Compared with that in the UFPM group, the red fluorescence intensity in the SMF group increased and the green fluorescence intensity decreased. The red/green fluorescent signal ratios in the control and UFPM cells were 1.109 ± 0.39 and 0.50 ± 0.09, respectively, whereas the signal in the SMF (1.12 mg/ml) + UFPM group was 0.6935 ± 0.22 (Figure 5B).

To determine whether the SMF-induced attenuation of mitochondrial dysfunction in UFPM-treated cells was followed by functional changes in energetics, the Seahorse assay was used. We found that SMF increased OCR levels markedly under basal conditions (UFPM control, 33.63 ± 2.11 pmol/min; 0.56 mg/ml SMF, 40.11 ± 3.09; Figure 5C) and increased proton leak (UFPM control, 6.073 ± 0.79; 0.28 mg/ml SMF, 9.87 ± 0.58; Figure 5D), ATP production (UFPM control, 25.18 ± 1.07; 0.28 mg/ml SMF, 30.68 ± 2.16; Figure 5E), and non-mitochondrial respiration (UFPM control, 31.34 ± 2.01; 0.56 mg/ml SMF, 39.61 ± 4.69: Figure 5F) compared with that in UFPM-exposed cells.

#### PCR Array

**FIGURE 6:**
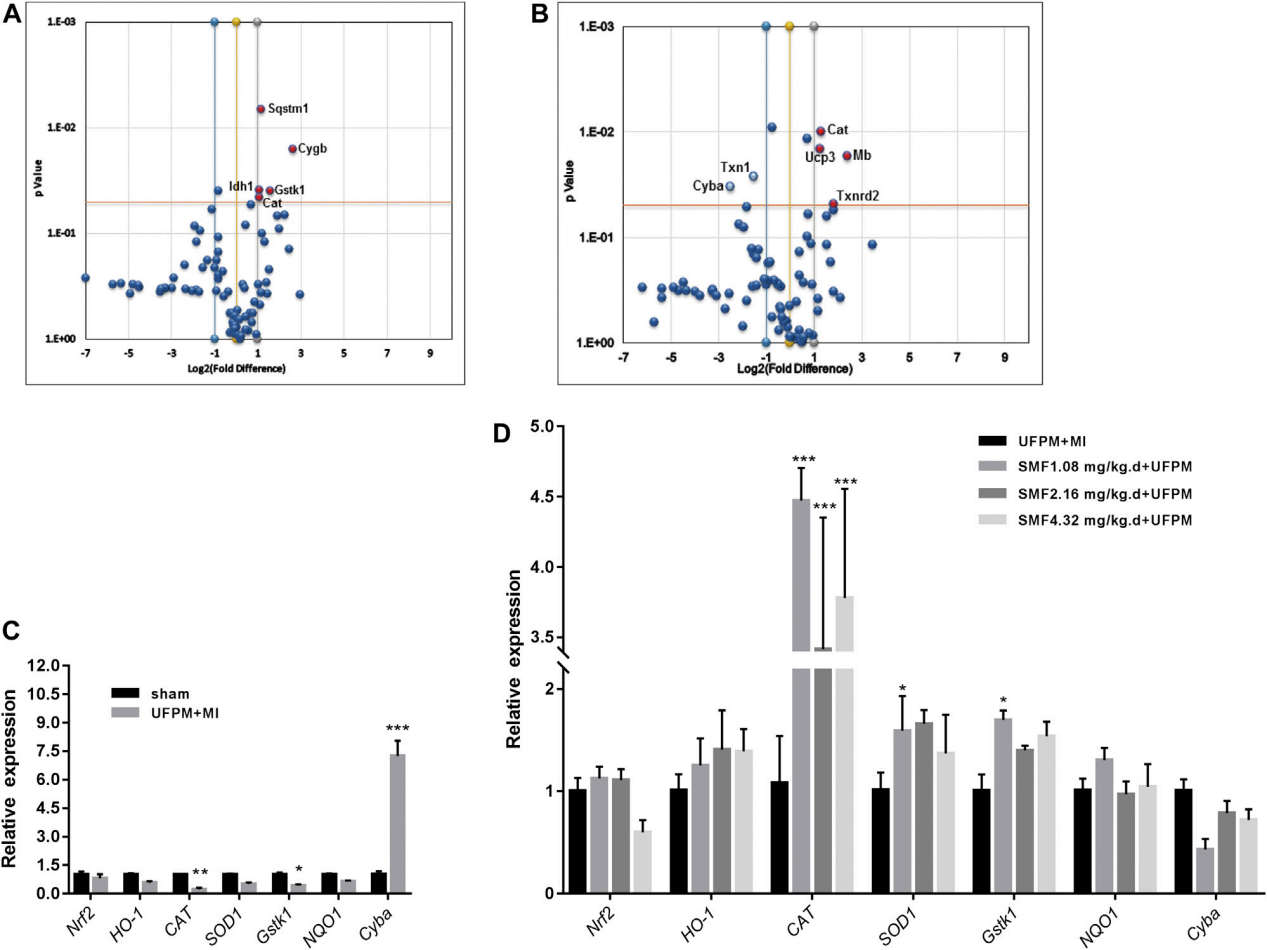
RT^2^ Profiler PCR array analysis of oxidative stress-related gene expression in the myocardial tissues of rats. **(A)** A scatter plot comparing the expression of 84 oxidative stress genes from rats exposed to UFPM gene expression in the sham controls. **(B)** A comparison of gene expression in rats pretreated with SMF before UFPM exposure to UFPM-exposed rats. Red solid circles identify genes with at least a 2-fold decrease **(A)** or a 2-fold increase **(B)** in expression. The central line represents unchanged gene expression and the boundaries represent the 2-fold cut-off value for changes in expression. **(C,D)** qRT-PCR validation of some oxidative stress genes. The histogram represents fold-changes in genes up- or downregulated in the UFPM group compared to the sham and SMF groups compared to the UFPM group.

Changes in the expression levels of several OS-associated genes were evaluated (Figures 6A,B). Of the 84 genes assayed in the array, the expression levels of two changed at least 2-fold (*Cyba* and *Ucp3*) and those of five changed 0.5-fold (*Cat*, *Gstk1*, *Cygb*, *Idh1*, and *Sqstm1*) in the UFPM-exposed MI rats compared with those in the sham control rats (Table 2). The expression levels of *Cat*, *Mb*, *Ucp3*, and *Txnrd2* were upregulated and the expression levels of *Txn1* and *Cyba* were downregulated in the SMF treatment group compared with those in the UFPM-exposed MI rats (*p* < 0.05; Table 3). The genes that exhibited opposite expression patterns in the drug and UFPM model groups were termed contrary genes (*Cat*, *Gstk1*, and *Cyba*). RT-qPCR was performed using these contrary genes to validate the results obtained by the PCR array analysis (Figures 6C,D). Trends in the expression levels of these genes confirmed that SMF treatment can maintain oxidative homeostasis.

**TABLE 2:** Expression of oxidative stress-associated genes in UFPM-exposed rats with MI.

Symbol	Description	Fold-change (2^−ΔΔCt^)	*p*-value
*Cat*	Catalase	0.47	0.0454*
*Gstk1*	Glutathione S-transferase kappa 1	0.34	0.0393*
*Cyba*	Cytochrome b-245, alpha polypeptide	2.15	0.0598
*Ucp3*	Uncoupling protein 3 (mitochondrial, proton carrier)	2.29	0.1005
*Cygb*	Cytoglobin	0.16	0.0159*
*Idh1*	Isocitrate dehydrogenase 1 (NADP+), soluble	0.48	0.0391*
*Sqstm1*	Sequestosome 1	0.45	0.007**

* and ** indicate significant differences from the sham group at *p* < 0.05 and *p* < 0.01, respectively. UFPM, ultrafine particulate matter; MI, myocardial ischemia.

**TABLE 3:** Expression of oxidative stress-associated genes after SMF treatment in UFPM-exposed rats with MI.

Symbol	Description	Fold-change (2^−ΔΔCt^)		*p*-value
*Cat*	Catalase		2.43	0.0100**
*Gstk1*	Glutathione S-transferase kappa 1		2.91	0.0627
*Cyba*	Cytochrome b-245, alpha polypeptide		0.18	0.0335*
*Ucp3*	Uncoupling protein 3 (mitochondrial, proton carrier)		2.40	0.0147*
*Mb*	Myoglobin		5.31	0.0171*
*Txn1*	Thioredoxin 1		0.34	0.0266*
*Txnrd2*	Thioredoxin reductase 2		3.57	0.0482*

* and ** indicate significant differences from the sham group at *p* < 0.05 and *p* < 0.01 vs. UFPM-exposed MI rats. UFPM, ultrafine particulate matter; MI, myocardial ischemia.

#### Involvement of the p38 MAPK, PI3K/AKT, and Nrf2/HO-1 Pathways

**FIGURE 7:**
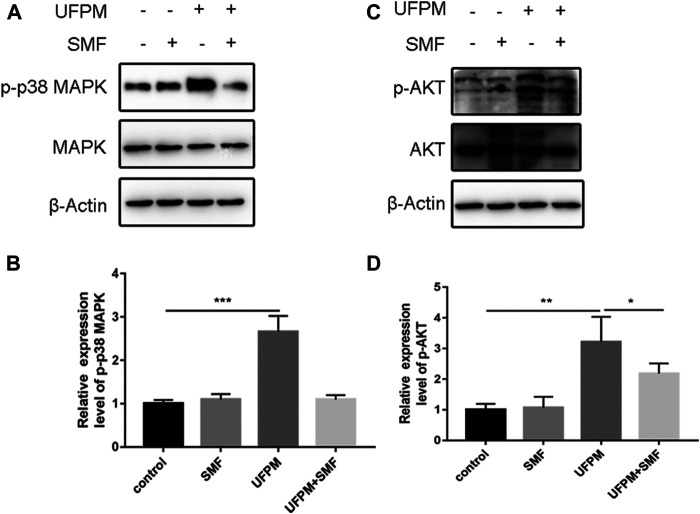
SMF inhibits UFPM-induced p38 MAP kinase and PI3K/AKT activation. H9C2 cells pre-incubated with either medium or SMF (1.12 mg/ml) for 24 h were stimulated with UFPM (50 mg/ml) for 6 h. The lysates from H9C2 were analyzed by western blotting using specific antibodies to **(A)** phospho-p38 MAP kinase (p-p38 MAPK) and **(B)** p-AKT. The cells were cultured with medium (*lane 1*), SMF (*lane 2*), UFPM (*lane 3*), and UFPM and SMF (*lane 4*). Fold increases in amounts of phosphorylated p38 MAP kinase proteins are indicated. The amounts of p-p38 MAPK and p-AKT were quantified using NIH Image Analyzer and are shown as the amounts relative to control cells. Three identical experiments were independently performed providing similar results.

We examined the effect of UFPM and SMF on p38 MAPK and PI3K/AKT signaling by measuring the expression levels of phosphorylated (p)-p38 MAPK and AKT in H9c2 cells (Figures 7A,C). The expression levels of p-p38 MAPK and p-AKT were significantly higher in the UFPM-treated group than in the untreated cells (Figures 7B,D; *p* < 0.001 and *p* < 0.01, respectively). Conversely, treatment with SMF significantly reduced p-p38 MAPK and p-AKT levels compared with those in the UFPM group (*p* < 0.05).

**FIGURE 8:**
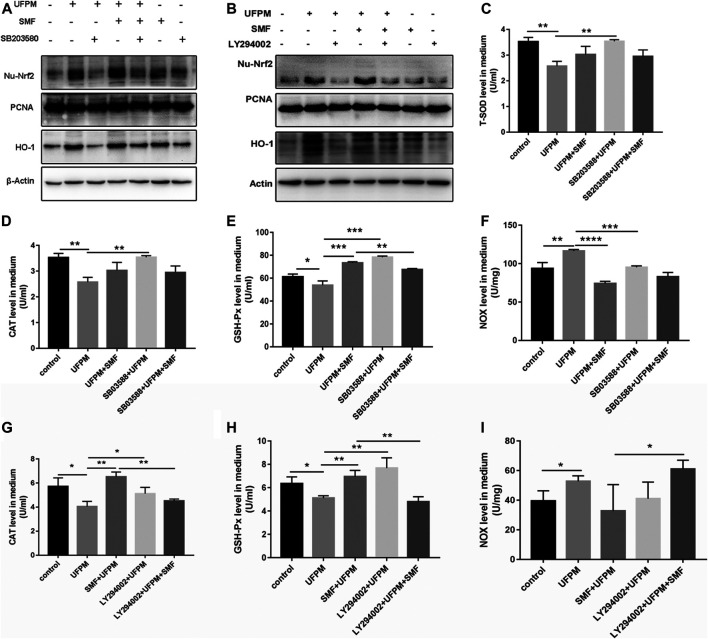
SB 203580, LY294002 and SMF inhibit UFPM-induced HO-1 and Nrf2 expression and T-SOD, CAT, NOX, and GSH-Px production. H9C2 pre-incubated with medium or SB 203580 (10 mM) for 60 min and LY294002 (50 μM) for 4 h were cultured with medium or SMF for 24 h, and then treated with or without UFPM (50 mg/ml) for 6 h **(A,B)** Western blot analysis of HO-1 and Nrf2 expression in cells. T-SOD **(C)**, CAT **(D,G)**, GSH-Px **(E,H)**, and NOX **(F,I)** concentrations in the culture supernatants were determined 6 h after UFPM exposure. The results are expressed as the mean ± SD of three different experiments. Asterisks indicate significant differences by *t*-test, *p < 0.05, **p < 0.01, ***p < 0.001.

Nrf2/HO-1 is a positive downstream pathway of MAPK and PI3K. We investigated the potential effects of SMF on Nrf2/HO-1 in H9c2 cells by western blot analysis. UFPM exposure resulted in a considerable increase in the protein expression of HO-1 and nuclear Nrf2 (*p* < 0.01; Figures 8A,B). SMF treatment further increased HO-1 and Nrf2. The effects of p38 MAPK (SB 203580) and PI3K (LY294002) inhibitors on Nrf2/HO-1 expression were evaluated in H9c2 cells. As shown in Figures 8A,B, UFPM + SB203580/LY294002 treatment significantly decreased HO-1 and nuclear Nrf2 expression levels compared with those in the UFPM-exposed group (*p* < 0.001). However, SMF treatment significantly reversed this decrease (*p* < 0.01 and *p* < 0.05, respectively). These results suggest that UFPM can act as a stimulating factor for the translocation of free Nrf2 into the nucleus to increase the expression of antioxidant-stress proteins, while SMF can promote this activation. However, inhibitors of p38 MAPK and PI3K prevented these effects.

The combination of UFPM and SB203580/LY294002 significantly increased the levels of T-SOD, CAT, GSH-Px and reduced the level of NOX in the medium compared to UFPM exposure without inhibitors (Figures 8C–I). In contrast, when SMF was added to UFPM and SB203580/LY294002 treatment, the level of GSH-Px in the medium decreased (Figures 8E,H), compared with that in the SMF and UFPM treatment (without the inhibitors) (*p* < 0.01). Furthermore, SMF + SB203580/LY294002 administration increased the levels of the NOX factors in UFPM-exposed H9c2 cells compared with SMF treatment alone (Figures 8F,I), suggesting a slight reversal in the effect of SMF on the expression of NOX factors. These findings indicate that SMF exerts antioxidative effects on UFPM-induced H9c2 cells *via* the PI3K/AKT and p38 MAPK pathways.

## Discussion

In this study, we showed that SMF treatment can protect cells against UFPM-induced myocardial injury and OS events both *in vitro* and *in vivo*. In addition, UFPM exposure induced ROS production, which activated the p38 MAPK/PI3K pathway, increased the expression levels of Nrf2/HO-1, and decreased the expression of antioxidative proteins. In contrast, SMF attenuated the UFPM-induced activation of MAPK/PI3K and increased the expression level of Nrf2, resulting in increased expression of HO-1 and antioxidative proteins.

In the present study, we analyzed the effect of acute exposure to UFPM on cardiovascular endpoints.The toxicity of PM can influence remote organs, includingthe heart (Du et al., 2016). The intratracheal instillation method used in the present study is regarded as an efficient method for the delivery of foreign substances (Driscoll et al., 2000). The dosage of UFPM used in the current study was similar to the dosage in previous studies involving rat models exposed to PM (Adams et al., 2019).

Our data show that acute exposure to UFPM causes significant myocardial damage characterized by increased myocardial infarct size, increased serum CK-MB and cTnT, and histopathological injury to the heart. In the present study, exposure to UFPM resulted in cardiomyocytedamage and mitochondrial dysfunction. In addition, the appearance of hyperplastic connective tissue and increased inflammatory cells suggested that UFPM exacerbates myocardial tissue injury leading to myocardial fiber edema. This may explain the low survival rate after MI in rats and the increased cardiovascular events accompanying urban air pollution. In addition, we examined cTnI, LDH, and CK-MB levels in serum, as specific markers of myocardial damage. The levels of these myocardial damage markers were higher in rats after UFPM exposure and MI compared with sham rats, supporting the involvement of UFPM in myocardial dysfunction. Published studies also demonstrate that PM2.5 exposure aggravates myocardial damage in hyperlipidemia rats (Wang et al., 2019).

The mechanisms by which UFPM induces cardiac damage remain unclear. UFPM may affect extra-pulmonary sites *via* inflammatory factors produced in the lung and secreted into the circulation (Thomson, 2019). Alternatively, UFPM may avoid the normal defenses of the lungs and gain access to the heart or brain (Du et al., 2016). However, one study showed that OS plays a critical role in the toxic mechanism of UFPM; UFPM may induce the formation or aggregation of free radicals (Yang et al., 2014).

In our study, UFPM exposure and MI significantly modified the OS status in rats, as indicated by increased MDA and NOX. Meanwhile, in our *in vitro* study, elevated levels of ROS were observed in the H9C2 cells exposed to UFPM, consistent with an imbalance in the oxidation/reduction observed in our *in vivo* study. A previous study demonstrated a significant difference in the expression levels of MDA, CAT, and GSH-Px in lung tissues of PM2.5-treated mice compared with saline-treated control mice (Huang et al., 2019). Similar increases in the levels of HO-1 and NOX in the lung and heart have been reported after particle exposure in rats; the differences were dependent on particulate size (Aztatzi-Aguilar et al., 2015). However, contrary to our findings, a recent study by OG Aztatzi-Aguilar reported a significant increase in SOD2 mRNA expression in the aorta of rats exposed to PM when compared to those in the filtered air group (Aztatzi-Aguilar et al., 2018). One explanation for this discrepancy is that the mRNA expression of SOD2 might have been elevated at the beginning of PM exposure in the previous study to increase the anti-oxidation ability of the myocardial cells and resist free radical damage.

The poor outcomes in response to MI in the current study may be due to the development of pulmonary artery hypertension (PAH) after UFPM exposure. PAH disturbs cardiac function. This is consistent with the findings of a previous study, which showed the general cytotoxicity of PM arising from different areas, possibly due to the similarities in the species used in the studies (Bein and Wexler, 2015). Several mechanisms may explain PAH-induced toxicity, including antioxidant defense impairment and OS (Holme et al., 2019). The antioxidant system attenuates oxidative injuries. Natural antioxidants have received increased attention over the past ten years. The effective biological activities of natural antioxidants are crucial during conditions that cause OS, such as air pollution (Zhang and Gao, 2014).

SMF is a known potent antioxidant agent; however, the protective effects of SMF on UFPM-induced cardiac damage/OS have not been reported. In the current study, SMF increased the survival rate in rats subjected to UFPM exposure and MI, suggesting that UFPM exposure-induced cardiotoxicity can be reversed by SMF. Cardiac dysfunction after UFPM exposure and MI impacted the survival rate and administration of SMF, which is beneficial in improving the heart function, improved the survival rate (Zhao et al., 2016).

Myocardial infarct size is a key prognostic factor in a wide range of adverse cardiovascular outcomes. Although there is little direct evidence for the induction of cardiac ischemia following exposure to UFPM in humans, experimental animal models have demonstrated an association between PM exposure and infarct size and/or MI. The results of the present study showed that SMF, administered 24 h after an acute single intranasal instillation of UFPM, significantly decreased the myocardium infarct size, indicating a significant recovery of myocardial function in rats with MI. This effect was further demonstrated by measuring LDH, CK-MB, and cTnT levels in the serum and the histopathological evaluation of the myocardial tissue. A previous study reported that three representative components in SMF showed significant protective effects on cardiac function after MI injury (Mo et al., 2015; Li et al., 2018).Thus, our results can be ascribed to specific components of SMF that interact in a complex way to regulate LDH, CK-MB, and cTnT activities. Further assessment is needed to connect the specific chemical components and biological activities.

As described above, UFPM disrupted OS status both in the MI rat model and in H9C2 cells. The disrupted OS status was signified by an imbalance of oxidative markers, mitochondrial membrane potential, and energy metabolism, while SMF prevented these outcomes. These results corroborate the findings of Zhu et al. (2019) (Zhu et al., 2019) demonstrating that the decreased levels of SOD, GSR, and CAT induced by H_2_O_2_ were upregulated following SMF treatment. The current study provides evidence that co-treatment with SMF in UFPM-exposed MI rats protected the heart from OS injury. Notably, the protective effects of SMF against UFPM-induced cardiotoxicity were not dose-dependent in the present study. This may be because differences in the selected doses in the current study were not large enough; hence, differences in the protective effects of SMF could not be detected.

Because SMF significantly attenuated UFPM effects in the serum and myocardial tissue, we hypothesized that multiple signaling pathways play a key role in the protective effects of SMF in rats exposed to UFPM and MI. To confirm this hypothesis, a herb-major hub-CVD disease-UFPM toxicity-pathway network was constructed to visualize the relationships among SMF herbs, major targets, CVD disease, and PM toxicity. Based on the integrated network-pathway analysis, SMF exerts antioxidant effects by regulating OS, which was identified as an important mechanism involved in UFPM toxicity and MI progression (Li et al., 2005; Kurian et al., 2016). OS was regulated *via* the PI3K/AKT signaling pathway, which was an important functional module with a high enrichment score in our study. In addition, the p38 MAPK pathway had a higher number of genes that were significantly affected but had low enrichment scores. We choose the p38 MAPK pathway based on the findings of previous studies. Austin Nguyen (2019) showed that inter-gene correlation could affect the enrichment score resulting in a pathway with lower expression values but more significantly affected genes (Austin Nguyen, 2019). Furthermore, PM causes a time- and dose-dependent increase in the phosphorylation of p38 MAPK in human pulmonary artery endothelial cells (Li et al., 2005). Marchini et al*.* (2016) showed that acute exposure to PM increased the levels of TNF-α, IL-6, and IL-1β, increased the ratio of active Caspase 3/pro-Caspase 3, and activated the NFκB pathway (Marchini et al., 2016; Li X. et al., 2017). TNF, CASP3, and NFKB1, which are among the key targets of SMF, are involved in p38 MAPK signaling. In addition, TNF, CASP3, and NFKB1 are targets of Ginseng radix et Rhizoma Rubra, Ophiopogonis Radix, and Schisandrae Chinensis Fructus, suggesting that these three targets play important roles in SMF action.

Because the OS response was also observed in the network analysis, the RT^2^ Profiler PCR array of myocardial tissue was designed to analyze a panel of genes related to the OS pathway. Our findings were in agreement with the detection of CAT in the serum of MI rats. A similar study also reported that SMF injection restored the mRNA expression of CAT after suppression by H_2_O_2_ (Zhu et al., 2019). Furthermore, the present study showed that SMF downregulated the gene expression of Cyba, which was consistent with a dramatic decrease in NOX levels in the serum of PM-treated MI rats. Cyba encodes a subunit of NOX. Thus, downregulation of Cyba reflected the decrease in myocardial OS after SMF treatment. NOX4 is highly expressed in cardiomyocytes and NOX4 activation causes cardiac dysfunction (Lassegue et al., 2012). Therefore, decreased myocardial NOX in the serum and myocardial tissue might help maintain myocardial function, supporting the protective effects of SMF in UFPM-induced oxidative injury. Additionally, the mRNA level of the antioxidant enzyme T-SOD was upregulated after SMF treatment in myocardial tissues of rats subjected to UFPM exposure and MI, in agreement with elevated serum T-SOD.

To further investigate the effects of SMF on the regulation of the OS pathways, we evaluated the p38 MAPK and PI3K/AKT pathways based on the net pharmacological data. UFPM induced the phosphorylation of p38 MAPK and AKT, decreased the levels of antioxidative factors such as GSH-Px, T-SOD, and CAT, and increased NOX production in H9C2 cells. These effects were prevented by SMF treatment. Nrf2 is activated under OS conditions and promotes the expression of numerous antioxidative enzymes such as T-SOD, CAT, and GSH-Px. Subsequently, protein expression levels of Nrf2/HO-1 were increased in response to SMF in H9C2 cells exposed to UFPM. However, Nrf2/HO-1 levels were also increased in the UFPM-exposed cells, which may be due to the specific components of UFPM and the stress response to acute UFPM exposure. Both SB 203580 and LY294002, specific inhibitors of p38 MAPK and PI3K, respectively, restored the inhibition of UFPM-induced NOX formation and the promotion of GSH-Px and CAT levels in SMF + UFPM-treated H9C2 cells. Meanwhile, SB 203580 and LY294002 decreased the expression levels of Nrf2/HO-1 in SMF + UFPM-treated H9C2 cells. Therefore, we speculated that p38 MAPK and PI3K/AKT might be involved in UFPM-induced OS. Our findings are in line with a previous report, which showed that the PI3K/AKT and p38 MAPK signaling pathways regulate HO-1 expression in RAW264.7 macrophages during anti-inflammatory responses (Li T. et al., 2017). Additionally, our data are consistent with previous studies, which showed the beneficial antioxidant effects of SMF on H_2_O_2_-induced oxidative injury (Zhu et al., 2019) and cerebral oxidative damage in rats (Ichikawa et al., 2003). Based on the findings that UFPM activated both p38 MAPK and PI3K/AKT, while their inhibitors and SMF inhibited these effects, we assume that the regulation of HO-1 expression and antioxidant proteins is controlled by the PI3K/AKT/p38 MAPK/Nrf2 pathways.

This study presents limitations. We have used the TTC staining method to measure the infarct size of MI rats. However, the normalized of area under ligation should be quality controlled by expressing infarct vs area at risk (AAR) by evan blue dye staning. We make sure that infarct size measurement will be normalized to AAR by evan blue dye staning in our following study. For cardiac injury analysis, flipped LDH combined with cTNT, CK-MB will be used in subsequent tudy rather than total LDH used in this study. In addition, an *in vitro* cell culture model related to MI and UFPM could be advantageous and the best way to present the signaling activation in animal model is to perform western blot analysis using heart tissue homogenate from animal treated with LY294002 or SB203580. Actually, we have tried to establish cell model about MI by hypoxia stimulation, however, most cells with hypoxia were dead after UFPM exposure due to the extremly harsh conditions. Considering that UFPM and MI can both produce the same toxicological effects, i.e., oxidative stress, therefore, we established the *in vitro* model which can reflect the oxidative stress injury by UFPM exposure to be maximally approximation to the true state of *in vivo* study. Meantime, we will perform western blot analysis using heart tissue homogenate from animal treated with LY294002 or SB203580 in our following study. Again, we will analysis mitochondrial respiration in isolated mitochondria from heart tissue of rat exposed to UFPM prior to LAD ligation for demonstrating mitochondrial respiration dysfunction directly.

In conclusion, the present study demonstrated that exposure to UFPM induced myocardial damage and mitochondria dysfunction, increased MDA, and decreased the levels of antioxidant factors in rats with MI. Pretreatment with SMF significantly relieved the cardiac-linked pathologies, inhibited the release of MDA and NOX, elevated the levels of antioxidant factors, and prevented the cardiovascular effects mediated, in part, by the PI3K/AKT/p38 MAPK/Nrf2 signaling pathway. Our results indicate that SMF is a potent antioxidant that protects against the deleterious effects of UFPM in the heart. SMF may be used to prevent and treat the cardiovascular events caused by UFPM exposure.

## Data Availability

The original contributions presented in the study are included in the article/Supplementary Material, further inquiries can be directed to the corresponding authors.
