# Supplementary material for: The Protective Effects of Shengmai Formula Against Myocardial Injury Induced by Ultrafine Particulate Matter Exposure and Myocardial Ischemia are Mediated by the PI3K/AKT/p38 MAPK/Nrf2 Pathway
Source: Front Pharmacol. 2021 Mar 8;12:619311. doi: 10.3389/fphar.2021.619311 (PMC7982744; doi:10.3389/fphar.2021.619311)
Supplement: Supplementary file 1 [file datasheet1.docx]

**Supplementary Figure 1. Chemical structure of active compounds of Shengmai formula.** A. Ginsenoside Rg1; B. Ginsenoside Re; C. Ginsenoside Rb1; D. Ruscogenin; and E. Schisandrin.

**Supplementary Figure 2. Liquid chromatography-mass spectrometry analysis of Shengmai formula (SMF).** (A) Total ion current (TIC) of the SMF sample (blue line) and solvent control (pink line) in positive ion mode and (B) in negative mode. (C) HPLC chromatogram of schisandrin identified from SMF. Retention time (RT) in minutes above the chromatographic peak denotes schisandrin in SMF.

**Supplementary Figure S3**. **The ingredient-target network of SMF.** The yellow and purple rectangle nodes represent ingredients, and the blue and red circle nodes represent targets. The purple rectangle and the red circle nodes indicate the key compounds and targets, invloving in the PI3K/AKT/p38 MAPK/Nrf2 pathway.

**Supplementary Figure 4. Evaluation of *in vivo* toxicity of ultrafine particulate matter (UFPM) and Shengmai formula (SMF). SMF suppresses UFPM-induced reactive oxygen species (ROS) production in H9C2 cells.** (**A**) Viability of H9C2 cells after 24 h of UFPM exposure. (**B**) Viability of H9C2 cells after 24 h of SMF exposure. (**C**) ROS detection in H9C2 cells using H_2_DCF-DA staining assay. H9C2 cells preincubated with or without SMF for 24 h were cultured with or without UFPM (50 mg/ml) for 24 h. The results were compared with those for the control cells and presented as p < 0.05 (*), p < 0.01 (**), p < 0.001 (***). The SMF data comparison with UFPM-exposed cells are presented as p < 0.05 (^#^), p < 0.01 (^#^), p < 0.001 (^###^).
