# Supplementary figures and images for: The Protective Effects of Shengmai Formula Against Myocardial Injury Induced by Ultrafine Particulate Matter Exposure and Myocardial Ischemia are Mediated by the PI3K/AKT/p38 MAPK/Nrf2 Pathway
Source: Front Pharmacol. 2021 Mar 8;12:619311. doi: 10.3389/fphar.2021.619311 (PMC7982744; doi:10.3389/fphar.2021.619311)

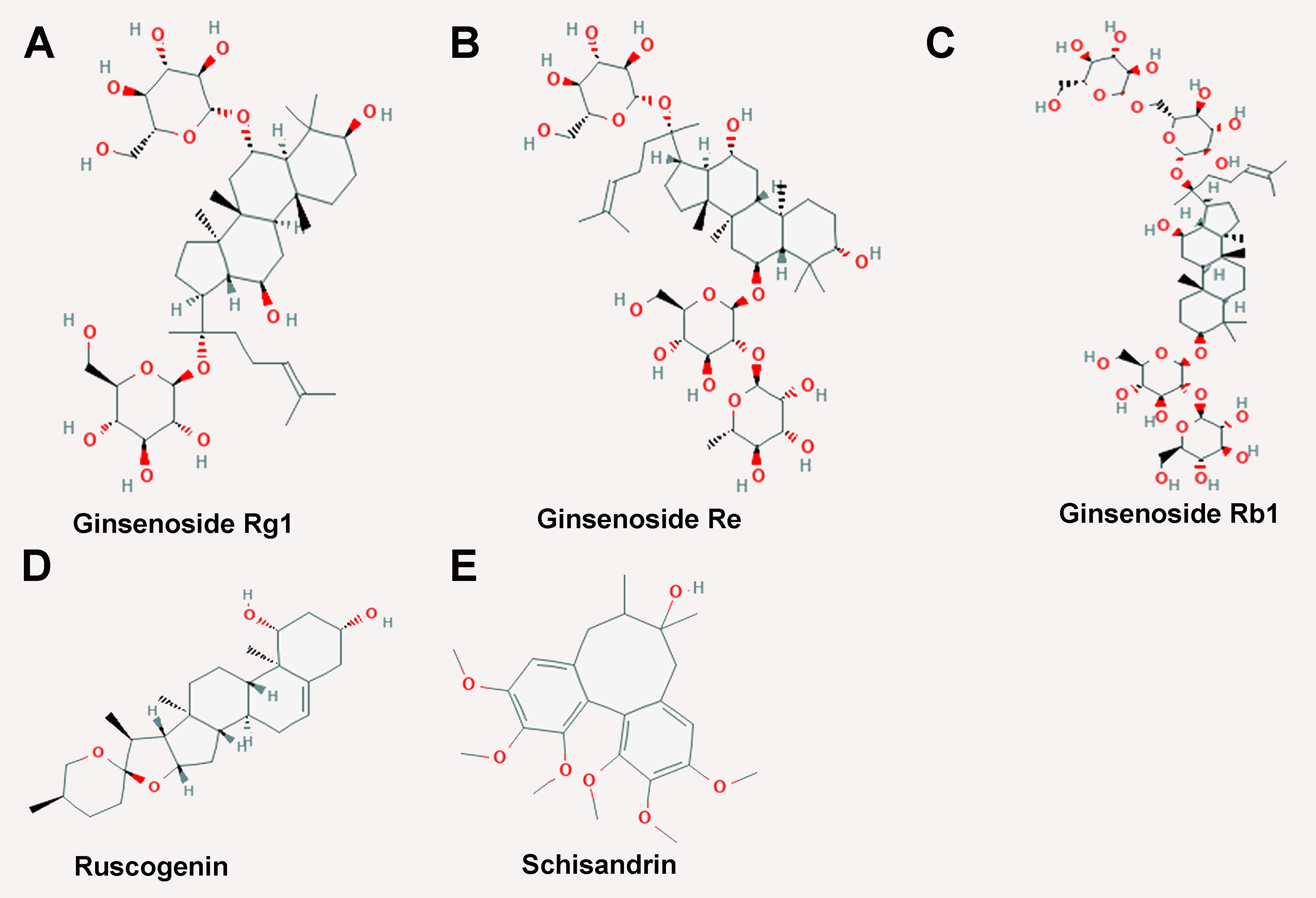

Supplement: Supplementary file 2 [file image1.tif]

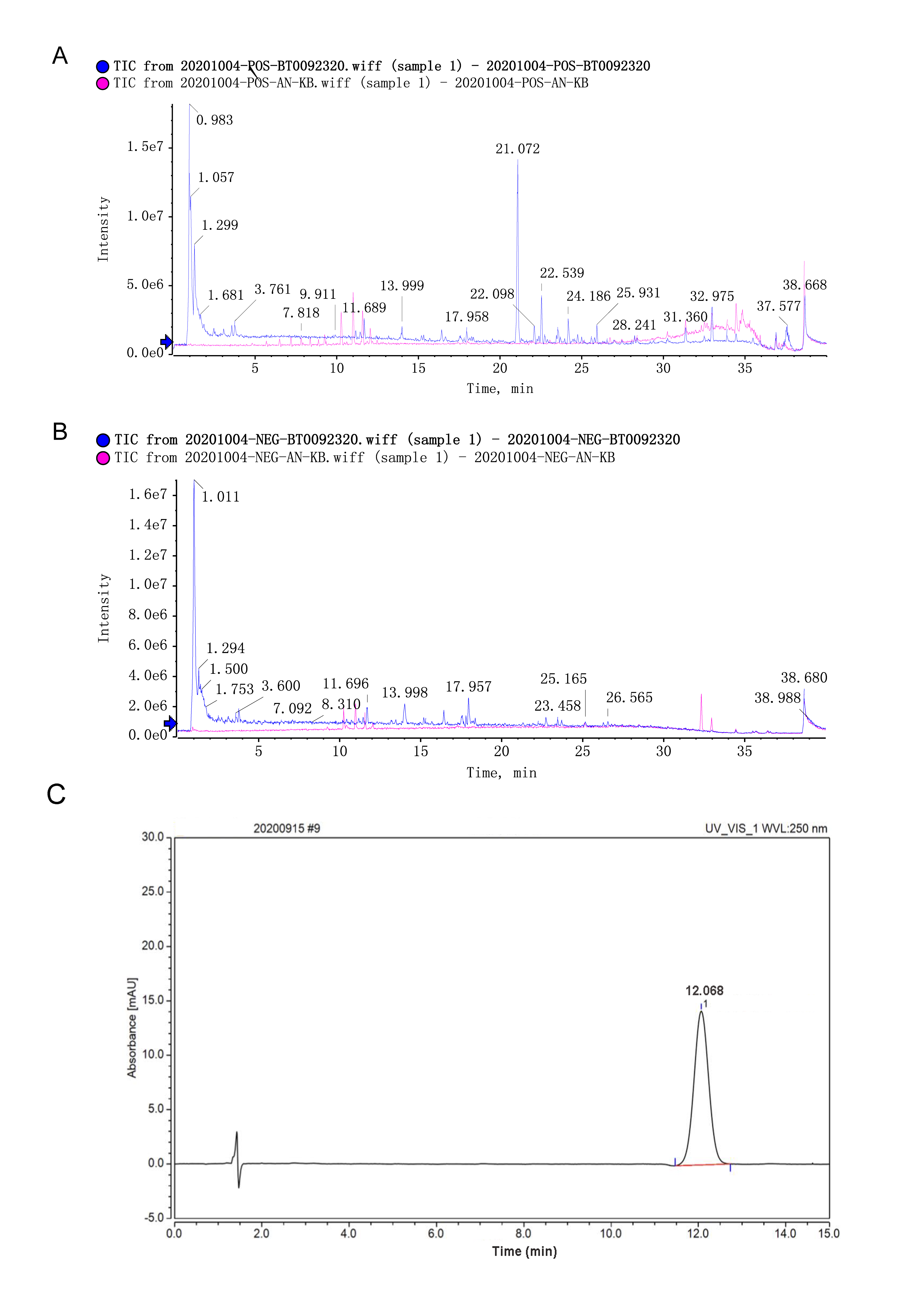

Supplement: Supplementary file 3 [file image2.tif]

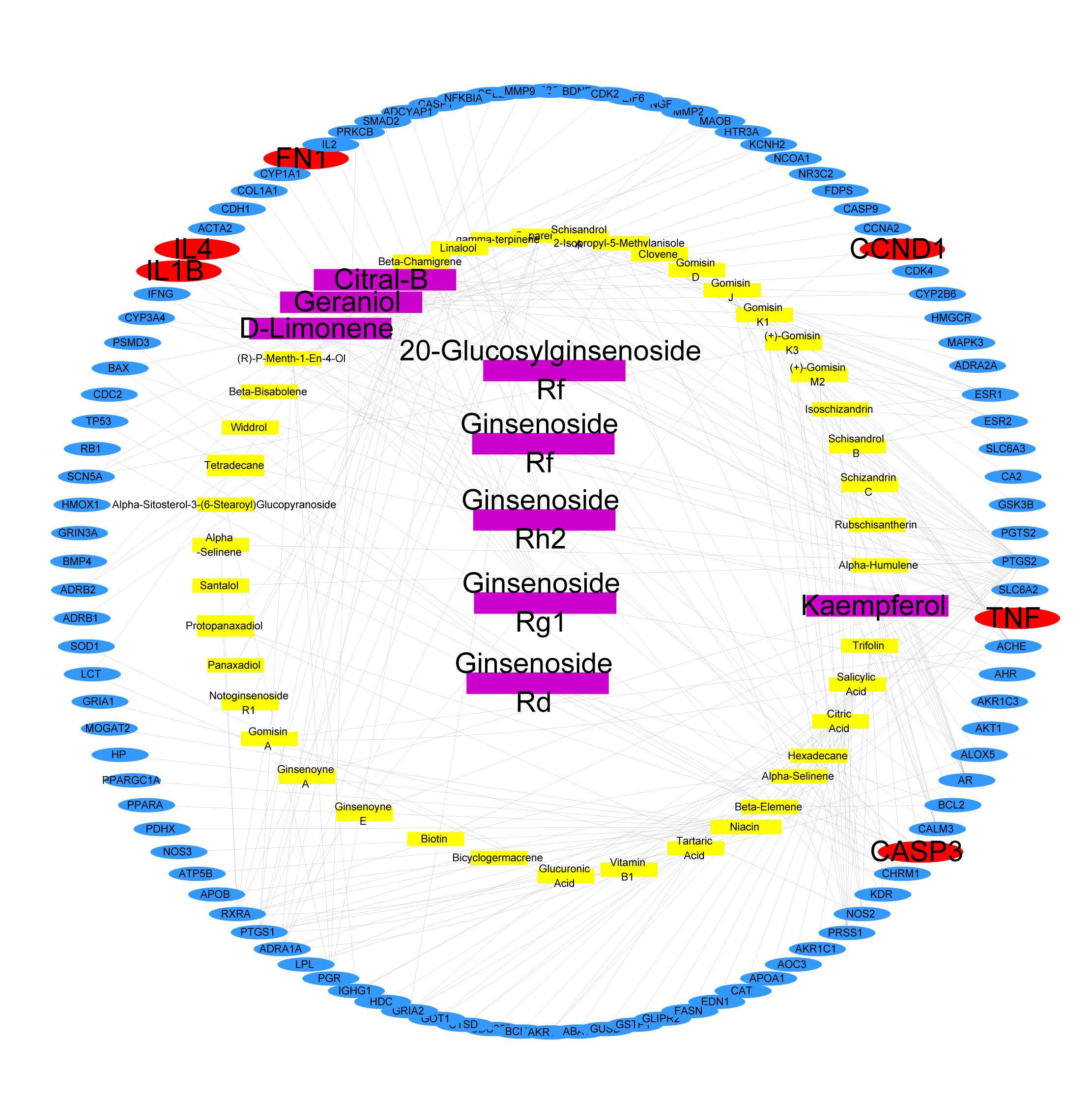

Supplement: Supplementary file 4 [file image3.tif]

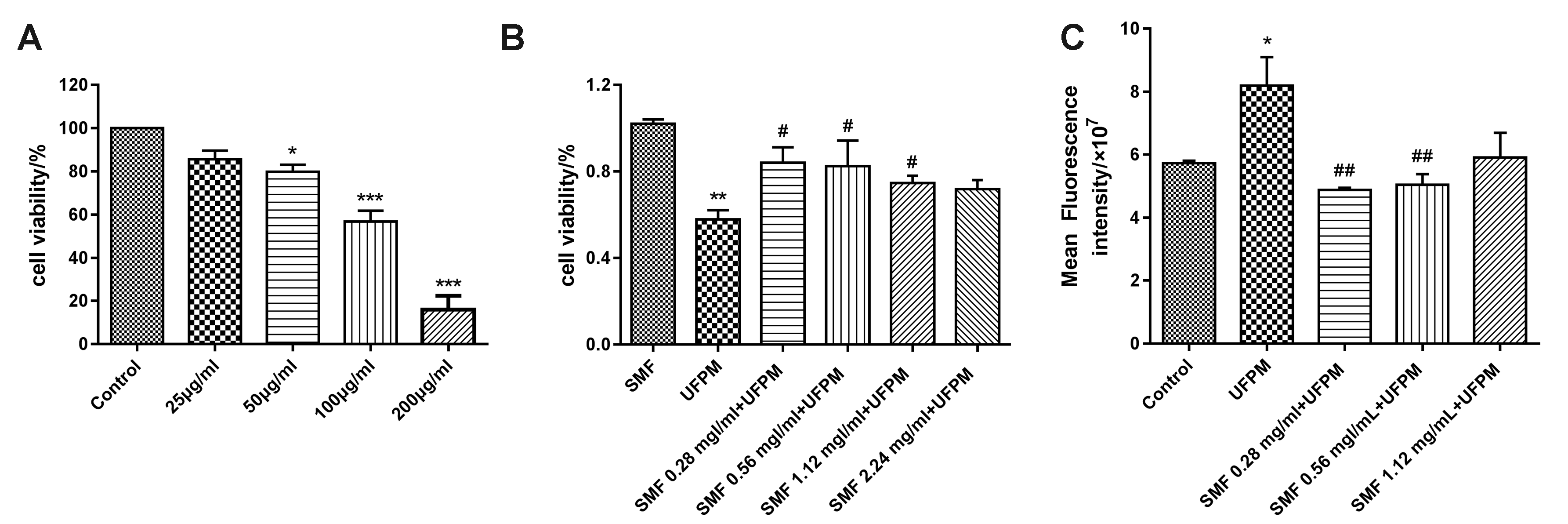

Supplement: Supplementary file 5 [file image4.tif]
