# Supplementary material for: The Protective Effects of Shengmai Formula Against Myocardial Injury Induced by Ultrafine Particulate Matter Exposure and Myocardial Ischemia are Mediated by the PI3K/AKT/p38 MAPK/Nrf2 Pathway
Source: Front Pharmacol. 2021 Mar 8;12:619311. doi: 10.3389/fphar.2021.619311 (PMC7982744; doi:10.3389/fphar.2021.619311)
Supplement: Supplementary file 7 [file table2.docx]

**Table S2 Detailed information of the three herbal ingredients in Shengmai formula**

| **Herbal ingredients** | **Original plants** | **Minimum concentrations of the dominating compounds based on pharmacopoeia** | **Reference** |
| --- | --- | --- | --- |
| Red Ginseng | It is the steamed and dried root of the cultivar *Panax ginseng* C. A. Mey. The drug is collected in autumn, washed clean, steamed, and dried. | It contains ≥0.25% of the total amount of ginsenoside Rg1 (C_42_H_72_O_14_), ginsenoside Re(C_48_H_82_O_18_), and ≥0.20% of ginsenoside Rb1 (C_54_H_92_O_23_), calculated with reference to the dried drug. | Pharmacopoeia of the People’s Republic of China, 2010, volume 1, P417-418. |
| Dwarf Lilyturf Tuber | Dwarf Lilyturf Tuber is the dried root tuber of *Ophiopogon japonicus* (Thunb.) Ker-Gawl. The drug is collected in summer, washed clean, sun-dried, piled up repeatedly until nearly dry, separated from rootlet, and dried. | It contains ≥0.12% of ruscogenin (C_27_H_42_O_4_), calculated with reference to the dried drug. | Pharmacopoeia of the People’s Republic of China, 2010, volume 1, P601-603. |
| Chinese Magnoliavine Fruit | It is the dried ripe fruit of *Schisandra chinensis* (Turcz.) Barll. The drug is known as “Bei Wuweizi” (Northern Magnoliavine Fruit). The drug is collected in autumn when ripe, dried in the sun or after steamed, and stalk and foreign matter are separated. | It contains ≥0.4% of schisandrin (C_24_H_32_O_7_). | Pharmacopoeia of the People’s Republic of China, 2010, volume 1, P782-784. |
