# Supplementary material for: The Protective Effects of Shengmai Formula Against Myocardial Injury Induced by Ultrafine Particulate Matter Exposure and Myocardial Ischemia are Mediated by the PI3K/AKT/p38 MAPK/Nrf2 Pathway
Source: Front Pharmacol. 2021 Mar 8;12:619311. doi: 10.3389/fphar.2021.619311 (PMC7982744; doi:10.3389/fphar.2021.619311)
Supplement: Supplementary file 8 [file table3.docx]

Supplement Table S3. List of primer sequences used for qRT-PCR analysis in this study. F: Forward primer. R: reverse primer

| **Genes** | **Primer sequences (5'to3')** | **PCR product length** |
| --- | --- | --- |
| **GAPDH** | F：TGCTGAGTATGTCGTGGAG | **288bp** |
|  | R：GTCTTCTGAGTGGCAGTGAT |  |
| **Nrf2** | F：ATATACGCAGGAGAGGGAAG | **222bp** |
|  | R：TCCCATCCTCATCACGTAAC |  |
| **HO-1** | F：GGGTCCTCACACTCAGTTTC | **228bp** |
|  | R：CCAGGCATCTCCTTCCATTC |  |
| **CAT** | F：TCACCGACGAGATGGCACACT | **174bp** |
|  | R：TGGAGAATCGGACGGCAATAGGA |  |
| **SOD1** | F：AGCAGAAGGCAAGCGGTGAAC | **216bp** |
|  | R：CGTCCTTTCCAGCAGCCACATT |  |
| **Gstk1** | F：GCAATCAGTTCGGACATCAGGAGA | **131bp** |
|  | R：CTTCTCACCATTCACCTCGCACTT |  |
| **NQO1** | F：TGGAAGAAGCGTCTGGAGACTGTC | **188bp** |
|  | R：ATCTGGTTGTCGGCTGGAATGGA |  |
| **Cyba** | F：CTGCCTCCATCAAGCCAAGATTCT | **146bp** |
|  | R：CCAATGCCTCCAGCCACACA |  |
